# Implementing mindfulness training for mental health professionals: Design and preliminary results of a controlled study

**DOI:** 10.1192/j.eurpsy.2026.11649

**Published:** 2026-07-31

**Authors:** J. Völflová Motlová, M. Janoušková

**Affiliations:** 1 Psychiatric Hospital Bohnice; 2 Division of Medical Psychology, Third Faculty of Medicine, Prague, Czech Republic

## Abstract

**Introduction:**

Mindfulness can be defined as the ability to be aware of what is happening in the present moment, non-judgmentally. Active mindfulness training for mental healthcare professionals can lead to stress and anxiety reduction and, conversely, increase their empathy, trait mindfulness, and self-compassion. These attributes contribute to the prevention and reduction of burnout syndrome. In the Czech healthcare environment, the potential benefits of an eight-week in-person mindfulness course for mental healthcare professionals have not yet been explored.

**Objectives:**

To examine the effects of an eight-week mindfulness course on perceived stress, empathy, self-compassion and resilience in healthcare professionals working in a psychiatric hospital.

**Methods:**

This is an intervention study. Participants will attend an eight-week Mindfulness-based stress reduction course led by a certified instructor. The program is offered to hospital employees as an employee benefit. Validated Czech versions of standardized scales (Self-Compassion Scale, Percieved Stress Scale, Five Factors Mindfulness Questionnaire, The Jefferson Scale of Empathy, Connor Davidson Resilience Scale) will be used. The research project is carried out with the approval of the Prague Psychiatric Hospital Ethics committee. The course capacity is for 13 employees (healthcare professionals) of the Psychiatric Hospital Bohnice. The control group will consist of employees who have applied for the course but cannot be admitted due to capacity of the group. Overall, the number of participants and the control group in total is estimated to be maximum 30 participants. Data will be collected through an on-line questionnaire administered via the REDCap platform, which enables the creation and secure management of online databases and surveys. Healthcare professionals will be sent a link to complete the questionnaire before the start of the course, after its completion, and several months later.

**Results:**

The data collection will begin in October 2025. We anticipate that participation in the mindfulness course will be associated with increased levels of empathy, mindfulness, self-compassion and resilience compared to the control group. These findings are expected to demonstrate the feasibility and acceptability of implementing structured mindfulness interventions for healthcare professionals in a Czech psychiatric hospital setting. Moreover, results could inform future workplace well-being programs and burnout prevention strategies.

**Conclusions:**

This study aims to contribute to the growing evidence on mindfulness training for healthcare professionals in Central Europe. Findings will provide insights into the potential role of mindfulness-based interventions as part of institutional support for staff well-being.

**Disclosure of Interest:**

None Declared

